# Antimicrobial activity of a quaternary ammonium methacryloxy silicate-containing acrylic resin: a randomised clinical trial

**DOI:** 10.1038/srep21882

**Published:** 2016-02-23

**Authors:** Si-ying Liu, Lige Tonggu, Li-na Niu, Shi-qiang Gong, Bing Fan, Liguo Wang, Ji-hong Zhao, Cui Huang, David H. Pashley, Franklin R. Tay

**Affiliations:** 1 School and Hospital of Stomatology, Wuhan University, Wuhan, China; 2 University of Washington, School of Medicine, Department of Biological Structure, Seattle, Washington, USA; 3 State Key Laboratory of Military Stomatology, Department of Prosthodontics, School of Stomatology, The Fourth Military Medical University, Xi’an, Shaanxi, China; 4 Tongji Hospital, Huazhong University of Science and Technology, Wuhan, China; 5 The Dental College of Georgia, Department of Endodontics, Augusta University, Augusta, Georgia, USA

## Abstract

Quaternary ammonium methacryloxy silicate (QAMS)-containing acrylic resin demonstrated contact-killing antimicrobial ability *in vitro* after three months of water storage. The objective of the present double-blind randomised clinical trial was to determine the *in vivo* antimicrobial efficacy of QAMS-containing orthodontic acrylic by using custom-made removable retainers that were worn intraorally by 32 human subjects to create 48-hour multi-species plaque biofilms, using a split-mouth study design. Two control QAMS-free acrylic disks were inserted into the wells on one side of an orthodontic retainer, and two experimental QAMS-containing acrylic disks were inserted into the wells on the other side of the same retainer. After 48 hours, the disks were retrieved and examined for microbial vitality using confocal laser scanning microscopy. No harm to the oral mucosa or systemic health occurred. In the absence of carry-across effect and allocation bias (disks inserted in the left or right side of retainer), significant difference was identified between the percentage kill in the biovolume of QAMS-free control disks (3.73 ± 2.11%) and QAMS-containing experimental disks (33.94 ± 23.88%) retrieved from the subjects (P ≤ 0.001). The results validated that the QAMS-containing acrylic exhibits favourable antimicrobial activity against plaque biofilms *in vivo*. The QAMS-containing acrylic may also be used for fabricating removable acrylic dentures.

There is increasing demand for orthodontic care worldwide. In the United States, approximately one-fifth of the adolescents and teenagers^1^, and up to 1% of young adults^2^ are receiving some form of the orthodontic treatment. According to the Medical Expenditure Panel Survey (2012) conducted by the U.S. Department of Health and Human Services, 24.4 million orthodontic procedures were performed on U.S children under the age of 20, and 5.3 million orthodontic procedures were performed on U.S. adults between the ages of 21 and 64 in 2009 alone^3^. One of the major challenges in orthodontic treatment is long-term stability; removable and fixed retainers are required to stabilise the aligned dentition and prevent post-treatment relapse^4^. Most removable retainers are constructed of polymethyl methacrylates (PMMA), which are held by metal clasps around the posterior teeth. Because removable retainers are worn in a moist intraoral environment with fluctuating pH for at least one year^5^, bacteria and fungi may accumulate on or within the retainers in the form of multi-species plaque biofilms that act as reservoirs of these microorganisms^6^,^7^,^8^. This may result in increased incidence of proximal dental caries^9^ or oral candidiasis^10^. Moreover, other opportunistic pathogens such as methicillin-resistant *Staphylococcus aureus* have been identified from orthodontic retainers^11^, which may potentially lead to local or systemic infection, particularly in orthodontic patients with complicated medical disorders^12^. For adult patients, oral microorganisms derived from removable acrylic appliances have been implicated in bacteria endocarditis, pneumonia, chronic obstructive pulmonary disease and gastrointestinal infection^13^. Production of volatile odoriferous compounds by colonised microorganisms also contributes to halitosis, which affects a person’s communication and psychological well-being^14^.

Microbial plaque biofilm accumulation on removable orthodontic appliances and retainers is usually controlled by mechanical and chemical means^11^,^15^,^16^. Despite their effectiveness, these procedures demand stringent patient compliance, which may not be readily achievable in those with restricted dexterity. Hence, incorporation of antimicrobial activity in orthodontic acrylic resin to achieve plaque biofilm reduction is highly desirable. Conventional PMMA-based antimicrobial approaches are based on leaching of antimicrobial agents of small molecular mass (e.g. chlorhexidine) into the intraoral environment, application of an antimicrobial coating on the surface of the material or incorporation of antibacterial silver nanoparticles into the PMMA resin^17^. Antimicrobial polymers are rapidly becoming a new class of biomaterials that can be functionalised and tethered to materials and kill microbes without releasing the biocides^18^,^19^. For methacrylates, anionic polymers have been created that can copolymerise with PMMA to create acrylic resins with permanent, non-leaching antimicrobial properties^20^.

Cationic polymers containing quaternary ammonium and phosphonium groups possess contact-killing antimicrobial activities^21^. An antimicrobial and antifungal cationic quaternary ammonium methacryloxy silicate molecule (QAMS) has been synthesised by sol-gel reaction between a tetraalkoxysilane and two trialkoxysilanes^22^. Containing a methacryloxy functional group and a long C-18 carbon chain, the QAMS molecule is soluble in MMA monomer and has been incorporated into PMMA orthodontic acrylic resin. The QAMS-copolymerised acrylic resin demonstrated improved fracture toughness without adversely affecting flexural modulus and strength of the orthodontic acrylic^23^. In previous *in vitro* studies, orthodontic acrylic resins containing 4–6% QAMS were found to possess *in vitr*o immediate diffusional as well as contact-killing antimicrobial properties when tested with *Streptococcus mutans*, *Actinomyces naeslundii* and *Candida albican*s^23^. To investigate the antimicrobial durability of the QAMS-containing acrylic resin, specimens were aged in water for 3 months prior to evaluation of their antimicrobial activities. Even after 3 months of water-ageing wherein any residual effects of diffusional kill would have been completely eliminated, the QAMS-containing orthodontic acrylic resin still possessed antimicrobial activities against single-species biofilms generated from the three microbes^24^. Antimicrobial polymers designed for biomedical applications should also be minimally cytotoxic to host tissues^18^. In a previous study, the viability of an odontoblast-like cell line derived from mouse dental papilla was examined by exposing these cells to QAMS-containing orthodontic acrylic resin. Results of the cell viability assays indicated that the QAMS-containing orthodontic acrylic resin is relatively non-cytotoxic^22^. The QAMS-containing orthodontic acrylic has received 510(K) clearance for marketing by the U.S. Food and Drug Administration (FDA)^25^. Nevertheless, clinical trials are lacking that demonstrate the *in vivo* antimicrobial potential of QAMS-containing orthodontic acrylic on multi-species biofilms.

Although various multi-species oral biofilm models have been developed and have contributed to the understanding of intraoral microbial adhesion and biofilm formation^26^,^27^,^28^, these models have drawbacks in that they are unlikely to replicate the variability and *in vivo* dynamics of plaque biofilms^29^,^30^. Apart from differences in structural characteristics between *in vitro* and *in vivo* biofilms, the presence of host defenses such as antimicrobial peptides derived from saliva^31^, is seldom taken into account in *in vitro* multi-species biofilm models^32^. More than 600 microbial species have been identified in the human oral microflora, of which approximately 280 species have been isolated in culture^33^. Thus, plaque biofilm profiles are unique among individuals, being modulated by different environmental factors^34^ as well as variable quorum sensing signals derived from adjacent microorganisms^35^,^36^. These confounding factors may temper the efficacy of antimicrobial polymers *in vivo*. Accordingly, the objective of the present randomised clinical trial was to determine the *in vivo* antimicrobial efficacy of the FDA-approved QAMS-containing orthodontic acrylic by using custom-made removable Hawley appliances that were worn intraorally by recruited subjects to create 48-hour multi-species plaque biofilms. Because of the anticipated high variability in the microbial composition of individual plaque biofilms, a split-mouth design was utilised to reduce inter-subject variability, with procedures taken to minimise unwanted carry-across effects^37^,^38^. The null hypothesis tested was that there is no difference in the antimicrobial activities between QAMS-free and QAMS-containing orthodontic acrylic resin on oral plaque biofilms grown *in vivo* in human subjects.

## Results

**Figure 1:**
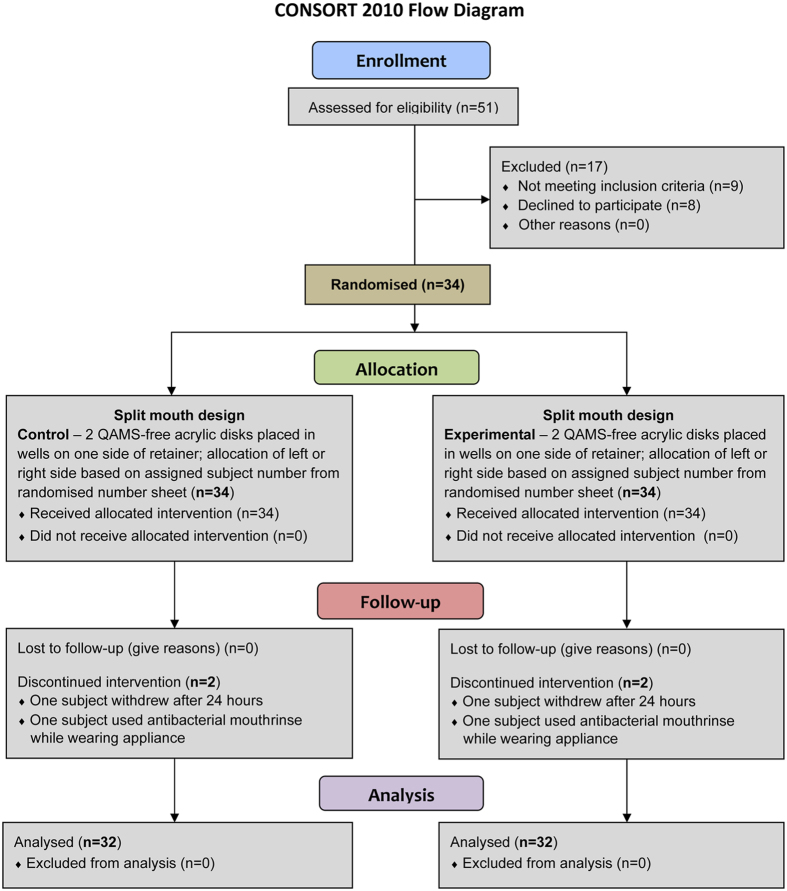
CONSORT flow diagram of subject randomisation and selection criteria.

A CONSORT flow diagram illustrating subject flow during the clinical trial is presented in Fig. 1. The entire study from subject recruitment to completion of data analysis spanned from December 2014 to September 2015. The recruitment period was from May to July, 2015. The 32 subjects who completed the trial consisted of 21 females and 11 males, with ages ranging from 22 to 56 years and with a median age of 32 years. The actual clinical part (primary outcome) of the trial was initiated between August 14 and 25, 2015, and included 48 hours of retainer wear as well as clinical follow-up after completion of the period of active appliance wear. Because biofilm examination had to be performed immediately upon retrieval of the acrylic disks, only 4 subjects were handled per working day. Data analyses for the study were completed in August 2015.

No harm to the oral mucosa, including redness, tenderness, swelling, ulcer or blister formation was observed in any of the 32 subjects after the 48-hour period of retainer well. In addition, no system harm induced by the QAMS-containing acrylic was reported by any of the 32 subjects up to the completion of the study in August 2015.

No significant difference in the overall biofilm thickness derived from the 32 subjects was observed between those deposited on the control acrylic disks (24.61 ± 5.33 μm) and those deposited on the experimental disks (27.45 ± 6.90 μm) (P = 0.070; 95% confidence interval [CI] for difference of means: 0.24–5.92). Percentage kills within the biovolume of *in vivo* plaque biofilms deposited on the acrylic disks are reported individually for each of the 32 subjects in Table 1. Significant differences between the control and experimental image stacks were observed for all subjects (P < 0.05 for all evaluations), indicating that there was negligible carry-across effect in the experimental design. Nevertheless, there was a wide range in the susceptibility of biofilms to killing by the QAMS-containing acrylic (mean and standard deviation of %kill =33.94 ± 22.88%; CI of mean: 8.611) compared to the QAMS-free acrylic (3.73 ± 2.11%; CI of mean: 0.762). Sixteen subjects had more than 25% mean %kill, while 16 subjects exhibited less than 25% mean %kill within the biofilms generated on the experimental acrylic disks.

There was no significant difference in the pooled %kill between control disks placed on the left side (4.27 ± 1.99%) and control disks placed on the right side (3.37 ± 2.17%) of the retainers (P = 0.115; 95% CI for difference of means: −0.63–2.43). Similarly, there was no significant difference in the pooled %kill between experimental disks placed on the left side (40.57 ± 26.55%) and experimental disks placed on the right side (28.49 ± 21.55%) of the retainers (P = 0.180; 95% CI for difference of means: −5.27–29.44). In the absence of allocation bias, data from control or experimental disks placed on the left and right sides of the retainers were pooled together to test the null hypothesis. Significant difference was identified between the pooled %kill in the biovolume of QAMS-free control disks (3.73 ± 2.11%) and QAMS-containing experimental disks (33.94 ± 23.88%) derived from the 32 subjects (P ≤ 0.001; Table 2).

**Figure 2:**
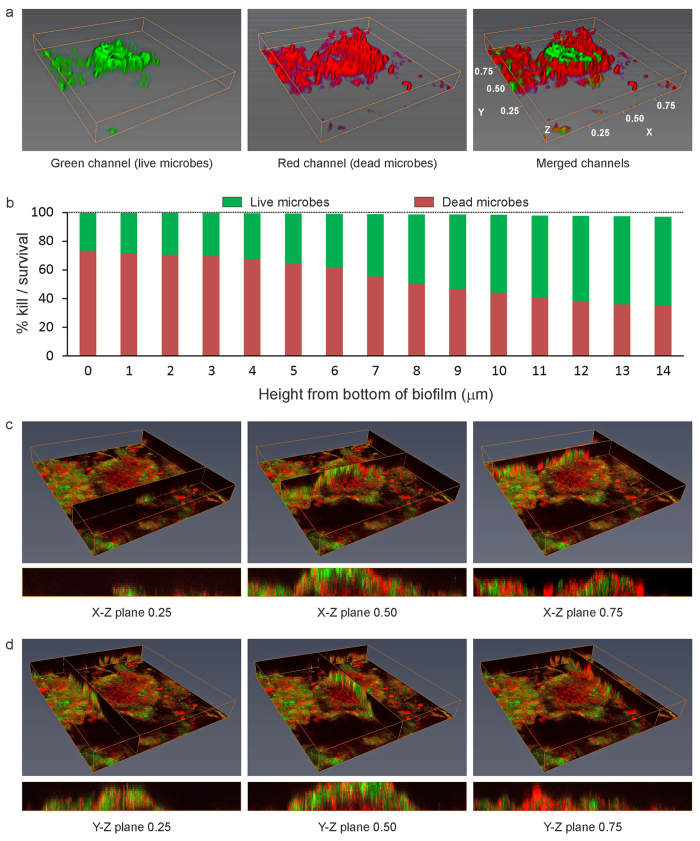
Representative BacLight-stained 48-hour biofilm grown on the surface of QAMS-containing acrylic resin (experimental disk) with >25% kill within the biovolume (Subject-3). (**a**) 3-D projection for the green, red and merged channels of the biofilm (the depth axis (Z-axis) was scaled-in to enhance presentation of the thickness of the biofilm). Green channel: live microorganisms; red channel: dead microorganisms. (**b**) Live/dead microorganism distribution from the bottom to the top of the biofilm, indicating the percentage of kill in different layers of the biofilms. (**c**) Perspectives of the biofilm from the X-Z plane of the 3-D projection (rectangular blocks beneath the projection) taken at one-quarter (0.25), one-half (0.50) and three-quarter (0.75) width of the Y-axis of the biofilm. (**d**) Perspectives of the biofilm from the Y-Z plane of the 3-D projection taken at 0.25, 0.50 and 0.75 width of the X-axis of the biofilm.

**Figure 3:**
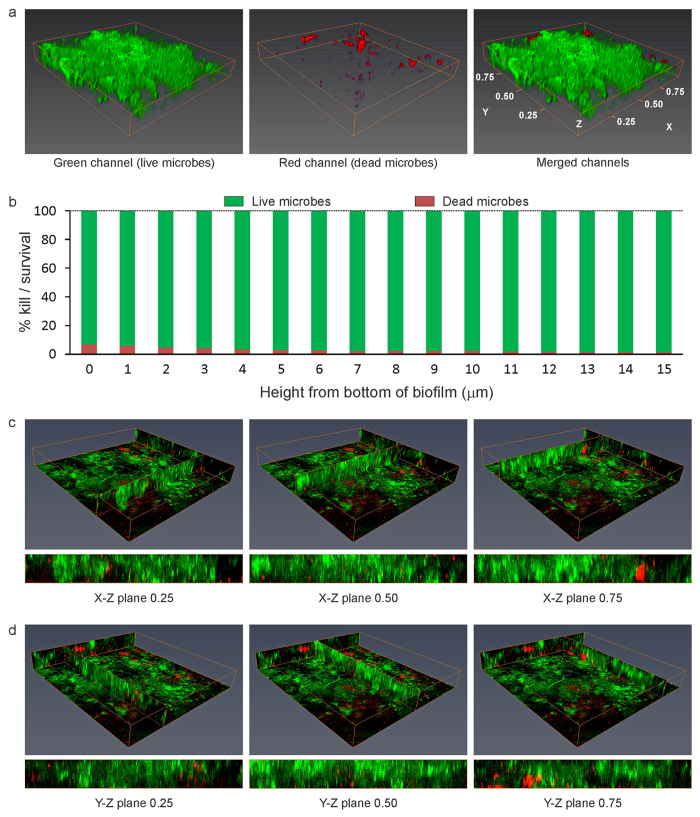
The corresponding BacLight-stained 48-hour biofilm grown on the surface of QAMS-free acrylic resin (control disk) of the subject presented in Figure 3 (Subject-3). (**a**) 3-D projection for the green, red and merged channels of the biofilm (Z-axis scaled-in to the same extent as Fig. 3). Green channel: live microorganisms; red channel: dead microorganisms. (**b**) Live/dead microorganism distribution from the bottom to the top of the biofilm, indicating the percentage of kill in different layers of the biofilms. (**c**) Perspectives of the biofilm viewed from the X-Z plane at 0.25, 0.50 and 0.75 width of the Y-axis. (**d**) Perspectives of the biofilm viewed from the Y-Z plane at 0.25, 0.50 and 0.75 width of the X-axis.

**Figure 4:**
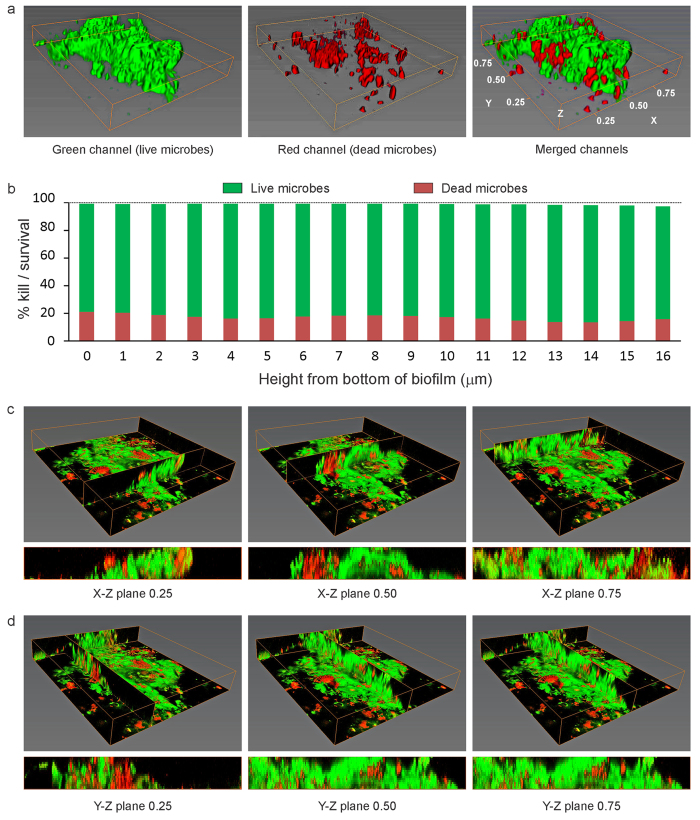
Representative BacLight-stained 48-hour biofilm grown on the surface of QAMS-containing acrylic resin (experimental disk) with <25% kill within the biovolume (Subject-25). (**a**) 3-D projection for the green, red and merged channels of the biofilm (Z-axis scaled-in to the same extent as Fig. 3). Green channel: live microbes; red channel: dead microbes. (**b**) Live/dead microbe distribution from the bottom to the top of the biofilm. (**c**) Perspectives of the biofilm viewed from the X-Z plane at 0.25, 0.50 and 0.75 width of the Y-axis. (**d**) Perspectives of the biofilm viewed from the Y-Z plane at 0.25, 0.50 and 0.75 width of the X-axis.

**Figure 5:**
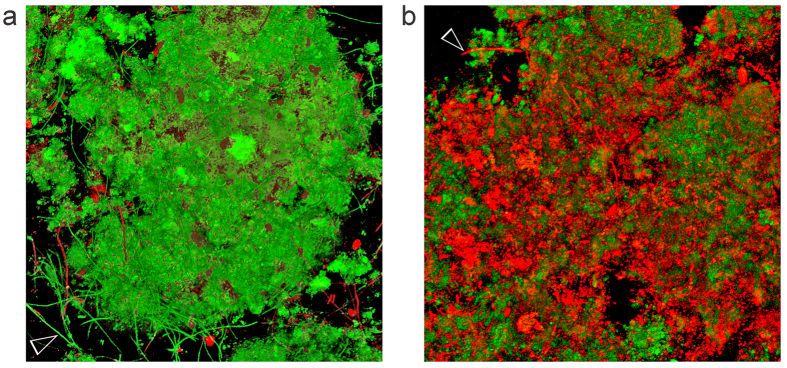
Example of plaque biofilms containing mixed bacterial-fungal components. (**a**) BacLight-stained CLSM merged image showing the presence of live (green) fungal hyphae (open arrowhead) within the biovolume of a biofilm taken from a control disk of one subject (subject 21). (**b**) The corresponding BacLight-stained CLSM merged image showing the presence of dead (red) fungal hyphae killed by the QAMS-containing acrylic (open arrowhead) from an experimental disk of the same subject (subject 21).

A representative example of BacLight-stained 48-hour plaque biofilm grown on the surface of QAMS-containing acrylic resin (experimental disk) with >25% kill within the biovolume is shown in Fig. 2 (movie of the stacked CLSM images in Supplementary Video S1); the corresponding example of stained 48-hour biofilm grown on the surface of QAMS-free acrylic resin (control disk) is shown in Fig. 3 (movie of the stacked CLSM images in Supplementary Video S2). A representative example of stained 48-hour biofilm grown on the surface of an experimental disk with <25% kill within the biovolume is shown in Fig. 4 (movie of the stacked CLSM images in Supplementary Video S3); the corresponding example of stained 48-hour biofilm grown on the surface of QAMS-free acrylic resin (control disk) is shown in Supplementary Fig. 1 (movie of the stacked CLSM images in Supplementary Video S4). Bacterial and fungal co-infections were identified in 38 out of the 256 plaque biofilms (8 biofilms/subject x 32 subjects) imaged from the control disks and 27 out of the 256 plaque biofilms imaged from the experimental disks. Figure 5 illustrates live and dead hyphae present in the plaque biofilms obtained from the control and experimental disks of one subject.

From the bar charts of live/dead microorganism distribution from the bottom to the top of the biofilms, as well as the Y-Z and X-Z vertical slices of the biofilms in Figs 2 and 4, it is evident that the presence of dead microorganisms was not limited to the bottom of the biofilms that were in contact with the acrylic resin surface. Thus, ancillary analyses of the biomass in the first 12 slices of the biofilms were performed. The results of biomass analyses (Table 2) indicated significant difference in pooled %kill for each slice between the QAMS-free control and QAMS-containing experimental disks (P < 0.00427 for all 12 analyses).

## Discussion

Randomisation and blinding are salient issues that affect the tenability of clinical trials. Randomisation ensures that all aspects between control and experimental groups are similar except for the intervention procedure. It eliminates selection bias and balances the groups with respect to known and unknown confounding variables. For clinical trials with a relatively small number of subjects, randomisation may be achieved by assigning numbers generated from a random number list to the intervention^39^. Blinding of subjects and investigators involved in clinical trials also minimises additional biases resulting from subjective inferences. The effects of intervention were found to be 14% lower in double-blind trials in which subjects, intervention providers and data collectors were blinded compared with similar trials there were not designated as double-blind^40^. In the present study, no significant difference between control and experimental disks placed on the left or right side of the retainer indicates that there was no allocation bias after randomisation and blinding were performed. A split-mouth experimental design removes much of the inter-subject variability, thereby increasing the power of the study compared to a whole-mouth parallel design. However, when there is a leakage of the intervention effect from one site to another site (carry-across effect), the study would be seriously handicapped in its capacity to provide unbiased assessment of the intervention. In the present study, leakage of antimicrobial monomers was minimised by ageing the polymerised acrylic disks in water for 3 months. The significant difference in %kill between the control and experimental disks in every subject is indicative of negligible carry-across effect in the study design. Based on these premises, it is surmised that the split-mouth design was a reasonable choice for generating results that lead to rejection of the null hypothesis that there is no difference in the antimicrobial activities between QAMS-free and QAMS-containing orthodontic acrylic resin on oral plaque biofilms grown *in vivo* in human subjects.

The combined use of SYTO-9 and propidium iodide (PI) fluorophores has been reported as a reliable method for evaluating bacterial viability^41^. Nevertheless, there are several limitations associated with the use of BacLight viability staining^42^,^43^, including: i) time-dependent bleaching of SYTO-9 signals, ii) differential affinity of SYTO9 for Gram-positive and Gram-negative bacteria, iii) background fluorescence and cross-signalling of one dye into another dye’s channel, and iv) intermediate states between ‘live’ and ‘dead’ bacterial cells. Bleaching of SYTO9 signals may result in underestimation of the total live bacterial cells. Because the observation of the acrylic disks in the present study took only 10–15 min per disk, the bleaching effect of SYTO-9 is unlikely to influence the acquired results. For evaluating a single bacterial species, the ineffectiveness of SYTO9 in staining some intact Gram-negative bacteria may adversely alter the vital bacterial cell count. Because both Gram-positive and Gram-negative bacteria are present in the oral environment, the overall microbial counts are large enough to neglect the bias caused by obstruction of the Gram-negative bacterial membrane. Understandably, different background signals have to be taken into consideration when calculating the exact signal intensity. The use of a split-mouth design to compare the live and death status of plaque biofilms facilitated in elimination of interference from background signals. Admittedly, some microbial cells exhibited yellow fluorescence instead of green/red fluorescence after SYTO9/PI staining due to the intermediate states between ‘live’ and ‘dead’ bacterial cells. In the present work, yellow signals that appeared in the CLSM images were eliminated to avoid such a bias. Thus, with the use of appropriate control, the BacLight viability staining method is generally well suited for studies on the effects of QAMS on *in vivo* multi-species plaque biofilms.

The SiQAC precursor from which QAMS is synthesised consists of three functional components. The first is a silane functionality which forms a network with other silanes via siloxane bridges produced by the hydrolysis and condensation reactions. The second is the positively-charged quaternary ammonium functionality, which enables the molecule to be attracted to the negatively-charged cell walls of microorganisms. The third is a long –C_18_H_37_ chain that achieves contact-killing by piercing through the cell walls of adherent microorganisms. As microorganisms come into contact with the surface of QAMS, they are first punctured by the long molecular chain. Electrostatic attraction between the positively-charged nitrogen atom in the quaternary ammonium functionality and the negatively-charged cell wall draws the microorganism closer to the –C_18_H_37_ chain, causing leakage of the cytoplasmic components that ultimately results in cell death. This contact-killing antimicrobial mechanism is incorporated into QAMS via sol-gel reaction with tetraethoxysilane as the linker molecule and 3-methacryloxypropyltrimethoxysilane as the methacryloxy-functionalised silane, and subsequently into PMMA while blending of QAMS with MMA monomer.

Large standard deviations were seen in the mean %kill of plaque biofilms deposited on QAMS-containing acrylic disks *in vivo* (Table 2). Although QAMS exhibits broad-spectrum antimicrobial activities against single-species bacterial and fungal biofilms *in vitro*, the *in vivo* sensitivity of biofilms to antimicrobial agents against device-related infections may not be as effective as that *in vitro*^44^. Moreover, some microbial strains may be more sensitive to antimicrobial agents than others^45^. This species diversity exists *in vivo* but not in multi-species biofilms cultured *in vitro*. Gram-positive bacteria have a comparatively thicker and more rigid peptidoglycan layer in their cell wall; deep, penetrative contact of the bacterial membrane by QAMS may be less likely to occur even under conditions of electrostatic attraction^46^. Species diversity within the biofilm may also improve its efficacy of survival under adverse environmental conditions via the growth of more antimicrobial-resistant microorganisms^47^, or alteration in the spatial distribution of more resistant microbial species to protect the less resistant members within the biofilm^48^. The resistance of plaque biofilms towards antimicrobial agents may also be altered by variations in quorum-sensing signals contributed by differential microbial constitutions. In mixed fungal-bacterial biofilms (e.g. Fig. 4, Movie SE-2), a wider range of less well known cross-kingdom interactions between the pathogens may be involved in increasing the virulence and antimicrobial resistance of the biofilm^49^. The quorum-sensing systems and interactions may be inhibited to different extents in the presence of QAMS. The plethora of factors that contribute to the survival and antimicrobial resistance of *in viv*o plaque biofilms may help explain why biofilms in some individuals are less sensitive to the effect of the QAMS-containing PMMA resin (Table 1).

Despite water-ageing of the QAMS-containing acrylic disks to enable leaching of non-polymerised and small incompletely-polymerised monomer components, vertical slices of the S-D biofilms in different X-Z and Y-Z planes clearly depicted that the presence of dead microorganisms was not limited to the bottom of the biofilms that was in contact with the acrylic resin surface (Figs 2C,D and 4C,D; Biomass data in Table 2). This phenomenon may be accounted for by the ability of microorganisms to undergo quorum-sensing signalling-induced programmed cell death in responses to environmental stresses^50^. Activation of the altruistic cell death mechanism proceeds via the release of pheromones such as the toxin component of toxin-antitoxin systems or bacteriocins (bacteriolytic molecules)^51^. This coordinated sacrificial lifestyle in the biofilm community enables elimination of non-competent members of the community to sustain survival of the remaining members or for re-colonisation. The advantages of programmed cell death in unicellular microorganisms are preservation of nutrients, as well as release of genomic materials into the biofilm matrix (i.e. extracellular DNA (eDNA)) for uptake and genetic exchange by the surviving, more competent members of that community. Taken together, advances in knowledge on the occurrence of apoptosis-like programmed cell death in bacteria and unicellular eukaryotes acquired over the past decade provides a rational explanation for the observation of 3-D killing within *in vivo* plaque biofilms that mimic the process of diffusional-killing by leached antimicrobial agents incorporated into polymers.

Co-infections by bacteria and fungi in biofilms have been implicated in enhanced host colonisation and virulence in urinary tract infections, ventilator-associated pneumonia and denture stomatitis^52^. For intraoral infections in denture wearers, inflammation of the oral mucosa is enhanced by the co-existence and interactions between *Candida albicans* and oral bacteria such as *Streptococcus*, *Actinomyces*, and *Fusobacterium* species to enhance adhesion of the fungus to the appliance surface^49^,^52^. In the presence trial, co-existence of bacteria and fungi could be identified in approximately 10% of all the plaque biofilms. Although antibacterial or antifungal agents have been separately incorporated into polymers, it is rare to find an antimicrobial polymer that possesses both antibacterial and antifungal properties. Hence, the ability of QAMS-containing acrylic to kill both bacteria and fungi (Fig. 5B) within the same plaque biofilm represents a valuable antimicrobial polymer for intraoral use for prevention of denture stomatitis.

Within the limits of the techniques employed in the present randomised clinical trial, it may be concluded that incorporation of 5 wt% QAMS in a PMMA-based orthodontic acrylic resin enables killing of microorganisms present in *in vivo* human plaque biofilms, without inducing harm to the oral mucosa after a 48-hour period of intraoral wear. Because this sustained kill was achieved by pre-ageing the processed acrylic in water prior to the clinical trial, it would be of interest in future clinical studies to examine if similar antimicrobial activities may be maintained through long-term wearing of acrylic resin intraoral appliances. Nevertheless, it should be pointed out that although a 3-D killing phenomenon was observed within the plaque biofilms, antimicrobial activity was the most potent along the base of the biofilm that was in contact with the surface of the acrylic resin. A gradual reduction in non-vital microbial biomass was observed with increasing distance from the base of the biofilm. Because the kill is not absolute, wearing of an intraoral acrylic appliance made of antimicrobial acrylic should not replace meticulous oral hygiene and extraoral cleansing of the appliance. The need for removable dentures and removable orthodontic appliances/retainers is unlikely to decrease in the near future. The material PMMA is still the best choice for fabricating denture bases and retainers due to its esthetic characteristics, high processing and polishing abilities, relining and rebasing possibility, and low cost. A potential extension of the antimicrobial technology is the incorporation of QAMS into full and partial acrylic dentures, which would be of significant preventive significance because of the long-term nature of the use of these intraoral removable prostheses.

## Methods

### Trial design

The protocol utilised in the present study was approved by the Ethics Committee of the School and Hospital of Stomatology, Wuhan University (approval number [2015A(01)]; Supplementary Fig. 2) and was registered under protocol ID ClinicalTrials.gov: NCT02525458 (date of registration 8/14/2015) according to the CONSORT 2010 statement^53^. The methods employed were performed in accordance with the approved guidelines. The trial is a double-blind (to the recruited subjects and to the data collectors and evaluators)^54^, split-mouth study (1:1 allocation ratio), conducted in a single centre in the Department of Prosthodontics of the School and Hospital of Stomatology, Wuhan University. Informed consent was obtained from all subjects.

### Sample size

Sample size was estimated based on the results derived from previous studies of *in vitro* killing of single-species biofilms by QAMS-containing acrylic^23^,^24^. For a 25% anticipated difference in means and a 35% anticipated difference in standard deviations between the control and experimental groups in a two-tailed t test, 32 subjects were required to achieve 80% power at α = 0.05.

### Participants

Announcements were posted within the School and Hospital of Stomatology, Wuhan University for recruitment of subjects over a 2-month period. The inclusion criteria for subject recruitment were:

1. Gender: male or female; Minimum age: 18; maximum age: 60

2. Healthy individual with no history or presence of a systemic disease

3. Absence of active caries or periodontal disease with pocket depths deeper than 4 mm

The exclusion criteria were:

1. Extensive gag reflex that precludes taking of an intraoral alginate impression

2. Presence of cleft palate that precludes the wearing of a Hawley retainer

3. Have been using an antimicrobial mouthwash prior to enrolment in the study

4. Have been taking antibiotics against infectious diseases in the 6 months preceding the study

Fifty-one volunteers were evaluated for eligibility, of which 9 failed to meet the inclusion criteria and 8 declined to participate. Thirty-four subjects (23 females, 11 males) were recruited to participate in the study.

### Interventions

Following the signing of a consent form, an alginate impression of the maxillary arch was taken for each recruited subject. A model of the maxillary arch was prepared in dental stone for construction of a removable Hawley retainer fabricated from heat-polymerised PMMA.

Sol-gel reaction was used for synthesising QAMS (CAS 1566577-85-4), as reported previously^22^,^23^. Briefly, tetraethoxysilane, 3-(triethoxysilyl)propyldimethyloctadecyl ammonium chloride (SiQAC) and 3-methacryloxypropyltrimethoxysilane (all from Sigma-Aldrich, St. Louis, MO, USA) were mixed in a 1:1:3 molar ratio and stirred at 200 rpm for 1 h. A stoichiometric amount of acidified water (16 moles; pH 2.5) was added to fully hydrolyse the monomers. Subsequent condensation of the silanol groups was conducted at pH 7.4 by adding sufficient 1 M NaOH to the previously-hydrolysed QAMS. The precipitate was heated at 100 °C and vacuum-stripped to remove the reaction by-products (i.e. water and alcohols), yielding a rubbery organically-modified silicate (ORMOSIL). Due to the antimicrobial activity endowed by the long, lipophilic –C_18_H_37_ alkyl chain^55^ of SiQAC^56^, an antimicrobial ORMOSIL with methacryloxy functional groups was produced that could copolymerise with methacrylate monomers.

A self-curing orthodontic acrylic resin system (Ortho-Jet; Lang Dental Manufacturing Co. Inc., Wheeling, IL, USA) was used for preparing the experimental and control test specimens. For preparation of the control disks, the MMA liquid component was mixed with the powder component in a mass ratio of 2:3. To prepare the experimental disks, QAMS was dissolved in the MMA liquid component, resulting in a QAMS-MMA comonomer blend with 5 wt% QAMS. The QAMS-MMA comonomer blend was mixed with the powder component in a mass ratio of 2:3. The doughs were packed into 6-mm diameter, 1-mm thick flexible moulds. A glass slab was placed on top of each packed dough to create a flat surface; the packed assembly was processed in a hydraulic pressure curing unit (Aurapres^®^, Lang Dental) to create dense, porosity-free acrylic disks. Sixty-eight control acrylic disks containing 0 wt% QAMS and 68 experimental acrylic disks containing 5 wt% QAMS were prepared.

To avoid carry-across effect in the split-mouth study design, freshly-prepared acrylic disks were immersed in water at 37 °C for 3 months prior to inserting into the retainers. This enabled unpolymerised monomer components to leach out of the processed acrylic. The rationale for using a 3-month ageing period was based on the results of bromophenol assay (for quantifying leached quaternary ammonium compounds in water)^24^. In that study, co-polymerisation of QAMS with the PMMA network resulted in only a minuscule amount of free QAMS molecules remaining within the polymerised acrylic after 3 months of water-ageing. The QAMS-containing acrylic retained its antimicrobial activities against single-species biofilms after 3 months of water-ageing^24^.

**Figure 6:**
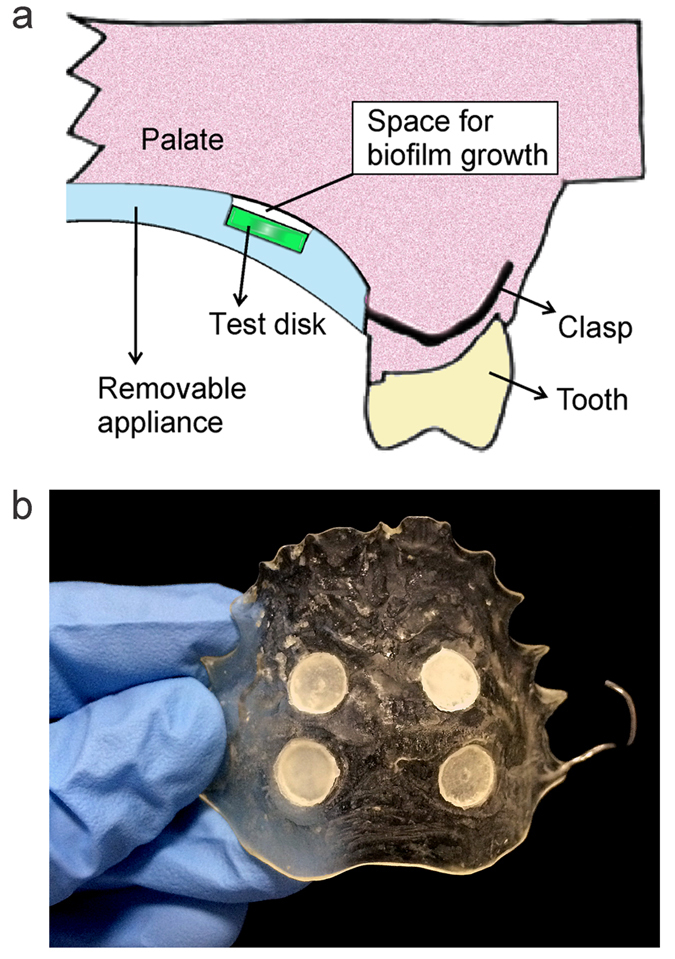
Appliance design. (**a**) Schematic illustrating the relationship between a test disk and its fitting well within the retainer. The disk surface to be examined was turned toward the palate but not in direct contact with the palate. Each well was protected from the oral cavity by orthodontic acrylic to prevent disturbance of the biofilm by the tongue. (**b**) Each appliance contained 4 wells to house two 6-mm diameter retrievable control disks and 2 similar diameter retrievable experimental PMMA disks. Allocation of the control disks to the left or right side of the retainer was decided by a randomised number sheet; the experimental disks were inserted subsequently into the wells on the contralateral side.

The control and experimental acrylic disks were disinfected with ultraviolet irradiation (UV-C, 300 kJ/cm^2^ for 10 min; Stratalinker^®^ UV Crosslinker, Model 2400; Stratagene, La Jolla, CA, USA) prior to insertion into the wells of the Hawley retainers. In the split-mouth design, two control QAMS-free acrylic disks were inserted into the wells on one side of the appliance, and two experimental QAMS-containing acrylic disks were inserted into the wells on the other side of the same appliance. Each control/experimental disk to be examined was inserted into the designated well, with the surface to be examined turned toward the palate but not in direct contact with the palate. This enabled the space between the disk and the palatal tissues available for growth of biofilm. Each well was protected from the oral cavity by acrylic to prevent disturbance of the biofilm through contact with the tongue (Fig. 6).

### Outcomes and follow-up

Thirty-four subjects wore the retainer continuously for 48 hours; each subject was instructed to maintain regular dietary behaviour while wearing the retainer during the trial. In addition, each subject was instructed to brush the teeth using a non-fluoride tooth paste and refrained from using antimicrobial mouthwash. Harms to the human subjects were minimised by examining the oral mucosa of each subject immediately after the retainer was removed for signs of redness, tenderness, swelling, ulcer or blister formation that is indicative of inflammation or allergic reaction. In the event that any of those episodes occurred, the involved subject was examined by a specialist in oral medicine to ensure that symptoms adequately subsided. Two subjects were discontinued from completion of the trial after 24 hours for the following reasons: one subject withdrew because of difficulty in wearing the retainer during eating, and the other used antibacterial mouthwash while wearing the retainer. Those two subjects were examined for potential harms to the intraoral tissues caused by wearing of the retainers; no harms were inflicted by the intervention.

Thirty-two subjects completed the trial. At the end of the designated 48-hour testing period, each subject was inspected for harms and instructed to resume routine oral hygiene procedures assumed by the individual prior to participating in the clinical trial. In addition, all subjects were instructed to report to the study facilitator (CH) at the School and Hospital of Stomatology, Wuhan University should any unusual systemic symptoms appeared after completion of the clinical part of the study. Each subject was then taken to the Confocal Laser Scanning Microscopy (CLSM) unit for retrieval of the control and experimental disks from the retainer. The biofilms formed on the disks were examined immediately for viability of the microbes using a CLSM at 40× magnification (LSM 510 META, Carl Zeiss GmbH, Jena, Germany). Each disk was stained using a live/dead staining kit (BacLight viability kit, Molecular Probes, Eugene, OR, USA). The kit consists of two nucleic acid stains: propidium iodide (PI) and SYTO-9. Live microbes were stained with SYTO-9 to produce green fluorescence, and microbes with compromised membranes were stained with PI to produce red fluorescence. The solutions were diluted with phosphate buffered saline (PBS, 0.01 M) according to the manufacturer’s instructions. Immediately after the disks were removed from the retainer, they were gently rinsed with PBS, incubated in the solutions in the dark at ambient temperature for 20 min and then rinsed with PBS. A 50-μL volume of 50 wt% glycerol was added to ensure that the specimens remained hydrated during CLSM examination.

The stained biofilms were examined using excitation wavelength of 488 nm for SYTO-9 and 568 nm for PI, respectively. Images of live and dead microbes within the biofilms were taken separately in the red and green channels or in the merged mode. For each acrylic disk, four Z-stacks (assortment of CLSM images representing a 3-dimenional section in a biofilm obtained at different levels of the z-axis) were obtained at a Z-step of 1 μm and a field size of 212 × 212 μm, beginning from the bottom of the biofilm that was in contact with the acrylic resin disk surface to the top of the biofilm. This yielded 8 Z-stacks for the control side and 8 Z-stacks for the experimental side for each subject. Segmentation and visualisation of images derived from CLSM imaging were performed using Amira 5.3.3. (Visage Imaging Inc., Andover, MA, USA).

### Randomisation

#### Sequence generation

Each recruited subject was randomly assigned based on a computer-generated randomisation number list^45^ to one of two groups: “1” for control disks in the left side of the retainer and “2” was for control disks in the right side of the retainer. The rationale for random allocation was to eliminate the effects of unilateral dietary or oral hygiene habits on the growth of biofilms.

#### Allocation concealment mechanism

The signed consent forms were kept in sequentially-numbered, opaque sealed envelopes containing the allocation sequence of the recruited subjects. The computer randomisation number list was kept in another sealed envelope by the researcher (SQG) in charge of inserting the disks into the wells of the order-numbered retainers. This researcher was not involved in clinical contact with the subjects, biofilm recording or data analysis. The stapled envelopes were opened and the allocation sequence was used for organising the image data files into control and experimental groups of the respective recruited subjects at the end of the trial.

#### Implementation and blinding

The allocation sequence (generated by SYL) was concealed from the recruited subjects, as well as researchers involved in enrolment and clinical contact with the subjects (CH), subject evaluation for harms following appliance removal (JHZ), CLSM examination (BF) and statistical analyses (LNN).

### Statistical methods

Merged images from each Z-stack derived from the plaque biofilms were analysed for the percentages of microbe killing (%kill) within the entire biovolume (volume covered by microbes in a 3-dimensional space), using the *bio*Image_L v2.1 image analysis software (Faculty of Odontology, Malmo University, Malmo, Sweden)^57^. For all analyses, data were examined for their normality (Shapiro-Wilk test) and equal variance (modified Levene test) assumptions prior to the use of parametric statistical methods. If those assumptions were violated, the data were nonlinearly-transformed prior to the use of parametric statistical methods. The Mann-Whitney rank sum test was used if those assumptions remained violated after nonlinear transformation.

The thickness of the biofilms deposited on the control and experimental disks of the 32 subjects was analysed with two-tailed Student t-test to see if there was significant difference in biofilm thickness between the two groups. To examine if a carry-across effect existed in the study design, the %kill within the biovolume of image-stacks acquired from biofilms in the control and experimental disks of each subject (n = 8) were analysed using two-tailed Student-t test, with “image stack” as the statistical unit. The mean %kill from image stacks derived from the control or experimental disks of each subject was employed for all subsequent analyses, with “subject” as the statistical unit. To examine if there was allocation bias when disks were allocated to the left or right side of the retainer, the means of the 8 Z-stacks from control disks placed on the left side of the retainers (designated as “1”; 14 subjects) were compared with the means of the 8 Z-stacks from control disks placed on the right side of the retainers (designated as “2”; 18 subjects), using two-tailed Student-t test. A similar analysis was performed for the experimental disks. To compare if there was significant difference in the %kill between QAMS-free and QAMS-containing acrylic resin (null hypothesis), the means of the 8 Z-stacks from control disks placed on both sides of the retainer were compared the means of the 8 Z-stacks from experimental disks placed on both sides of the retainer (n = 32 for both groups), using two-tailed Student-t test. For all analyses, statistical significance was preset at α = 0.05.

The %kill within the biomass (area covered by microbes in a 2-dimensional space) from each slice of the Z-stack were also analysed (32 subjects for both control and experimental groups) in light of the observation that killing of microorganisms in the biofilms was not limited to contact (surface) killing. Because biofilms vary in thickness, only the first 12 slices (from bottom to top) of the Z-stacks (present in all biofilms) were analysed individually by two-tailed Student t-tests. Due to the correlated nature of the data, Dunn–Šidák adjustment was used to control the family-wise error rate for testing 12 hypotheses using the same biofilm specimens (chance of finding one or more significant differences =45.96% in the absence of adjustment). Accordingly, only a P value < 0.00427 was considered significantly different between the QAMS-free and QAMS-containing acrylic resin in each respective slice.

## Additional Information

**How to cite this article**: Liu, S.-y. *et al.* Antimicrobial activity of a quaternary ammonium methacryloxy silicate-containing acrylic resin: a randomised clinical trial. *Sci. Rep.* **6**, 21882; doi: 10.1038/srep21882 (2016).

## Supplementary Material

Supplementary Information

Supllementary video S1

Supllementary video S2

Supllementary video S3

Supllementary video S4

## Figures and Tables

**Table 1:** Percentage kill within the biovolume of *in vivo* plaque biofilms deposited on acrylic disks from enrolled human subjects.

Assigned subject number	Disk allocation of control disk^†^	Control side (%)* [n = 8 biofilms]	Experimental side (%)* [n = 8 biofilms]	P value
1	left	4.25 ± 2.22	61.87 ± 19.88	≤0.001
2	left	2.69 ± 1.55	11.85 ± 8.80	0.039
3	left	4.88 ± 3.66	76.60 ± 7.98	≤0.001
4	right	0.66 ± 0.29	57.96 ± 17.63	≤0.001
5	left	8.97 ± 2.70	55.75 ± 12.84	≤0.001
6	left	2.74 ± 1.66	80.90 ± 9.57	≤0.001
7	left	3.45 ± 1.49	36.54 ± 6.87	≤0.001
8	right	3.94 ± 2.30	16.26 ± 7.91	≤0.001
9	right	4.59 ± 4.03	32.19 ± 22.15	0.049
10	left	4.09 ± 1.35	24.45 ± 14.99	0.004
11	left	4.13 ± 1.04	25.63 ± 15.41	0.003
12	right	1.96 ± 1.01	12.72 ± 7.84	0.008
13	right	1.41 ± 1.05	8.48 ± 5.91	≤0.001
14	right	4.61 ± 2.31	51.93 ± 7.91	≤0.001
15	right	1.86 ± 1.05	15.71 ± 7.19	≤0.001
16	right	2.03 ± 1.30	18.27 ± 12.70	≤0.001
18	left	7.08 ± 3.10	33.23 ± 17.00	≤0.001
19	right	4.54 ± 4.02	13.70 ± 8.62	0.003
20	left	5.94 ± 4.43	81.59 ± 12.44	0.004
21	right	2.57 ± 1.08	65.63 ± 12.59	≤0.001
22	left	4.67 ± 3.41	44.19 ± 11.37	≤0.001
23	left	1.57 ± 2.86	11.04 ± 6.69	≤0.001
24	right	1.27 ± 0.81	13.00 ± 5.07	≤0.001
25	right	6.26 ± 1.47	16.34 ± 8.28	≤0.001
26	right	2.84 ± 1.62	12.27 ± 7.42	≤0.001
27	right	5.60 ± 3.85	78.73± 8.60	≤0.001
28	right	3.66 ± 1.00	42.81 ± 7.95	≤0.001
29	left	3.00 ± 1.91	12.59 ± 4.11	≤0.001
30	right	9.24 ± 6.72	27.93 ± 8.41	≤0.001
32	left	2.35 ± 0.91	11.81 ± 6.11	0.001
33	right	1.93 ± 1.89	18.10 ± 10.89	0.002
34	right	1.63 ± 2.14	10.79 ± 4.79	≤0.001

^**†**^A randomised number sheet was used to decide allocation of the control disks to the left or right side of the retainer appliance. Allocation of the control disks to the right side of the appliance inferred that the experimental disks would be inserted in the left side of the appl**i**ance, and vice versa. Accordingly, there were 17 subjects with control disks inserted in the left side of the retainer and experimental disks inserted on the right side of the appliance, and 17 subjects with control disks inserted in the right side of the retainer and experimental disks inserted on the left side of the appliance. Two subjects with controlled disks allocated to the left side of the appliance were discontinued from intervention during the follow-up phase, resulting in 18 subjects with control disks inserted in the left side of the retainer and 14 subjects with control disks inserted in the right side of the retainer.

*Values are means ± standard deviations (in percentages).

**Table 2:** Percentage kill within plaque biofilms deposited on acrylic disks *in vivo*.

Parameter		Control side* (n = 32 subjects)	Experimental side* (n = 3 subjects)	P value^†^	95% CI^‡^ for difference of means
Biovolume		3.74 ± 2.11	33.94 ± 23.88	≤0.001	21.74–38.68
Biomass**	0 μm	7.45 ± 3.54	29.40 ± 11.01	≤0.001	17.86–26.04
	1 μm	6.84 ± 3.17	28.54 ± 10.83	≤0.001	17.71–25.69
	2 μm	6.41 ± 2.93	27.47 ± 10.57	≤0.001	17.19–24.94
	3 μm	5.95 ± 2.78	26.52 ± 10.50	≤0.001	16.74–24.41
	4 μm	5.46 ± 2.78	25.63 ± 10.44	≤0.001	16.37–23.98
	5 μm	5.05 ± 2.40	24.38 ± 9.66	≤0.001	15.82–22.85
	6 μm	4.93 ± 2.35	23.11 ± 9.01	≤0.001	14.89–21.47
	7 μm	4.91 ± 2.63	21.91 ± 8.69	≤0.001	13.79–20.21
	8 μm	4.32 ± 2.60	19.98 ± 7.89	≤0.001	12.72–18.59
	9 μm	4.42 ±3.06	19.63 ± 7.43	≤0.001	12.28–18.14
	10 μm	4.30 ± 3.23	18.78 ±7.79	≤0.001	11.35–17.61
	11 μm	4.51 ± 2.66	18.94 ± 9.39	≤0.001	10.57–18.18

*Values are means ± standard deviations (in percentages).

**Numbers represent distance from the bottom of biofilms. Because biofilms have different thickness, only slice levels that were present in all biofilms derived from the control side and the experiment side were statistically analysed.

^**†**^Dunn–Šidák correction was used to control the family-wise error rate for testing 12 hypotheses (i.e. 12 slices within the Z-stacks) using the same biofilm specimens. Accordingly, only a P value < 0.00427 was considered significantly different between the control side and the experimental side.

^**‡**^Confidence interval.
