# Supplementary material for: Antimicrobial activity of a quaternary ammonium methacryloxy silicate-containing acrylic resin: a randomised clinical trial
Source: Sci Rep. 2016 Feb 23;6:21882. doi: 10.1038/srep21882 (PMC4763235; doi:10.1038/srep21882)
Supplement: Supplementary Information [file srep21882-s1.pdf]

Antimicrobial activity of a quaternary ammonium methacryloxy silicate-containing acrylic resin: a randomised clinical trial

Si-ying Liu[1], Lige Tonggu[2], Li-na Niu[3]*, Shi-qiang Gong[4], Bin Fan[1], Liguo Wang[2], Ji-hong Zhao[1], Cui Huang[1]*, David H. Pashley[5], Franklin R. Tay[5]*

Supplementary Information

1. Supplementary Figure S1

2. Supplementary Video S1: 3-D reconstruction of a plaque biofilm with > 25% kill, taken from the experimental side (QAMS-containing acrylic disk) of subject 3. A higher resolution of the video is available at https://www.youtube.com/watch?v=81A-LvK5teo&feature=youtu.be.

3. Supplementary Video S2: 3-D reconstruction of a plaque biofilm taken from the corresponding control side (QAMS-free acrylic disk) of subject 3. A higher resolution of the video is available at https://youtu.be/GctXl1PfQ6E.

4. Supplementary Video S3: 3-D reconstruction of a plaque biofilm with < 25% kill, taken from the experimental side (QAMS-containing acrylic disk) of subject 25. A higher resolution of the video is available at http://youtu.be/YvbdYeX6RoM.

5. Supplementary Video S4: 3-D reconstruction of a plaque biofilm taken from the corresponding control side (QAMS-free acrylic disk) of subject 25. A higher resolution of the video is available at http://youtu.be/RbLNT0m7AHc.

Supplementary Figure S1

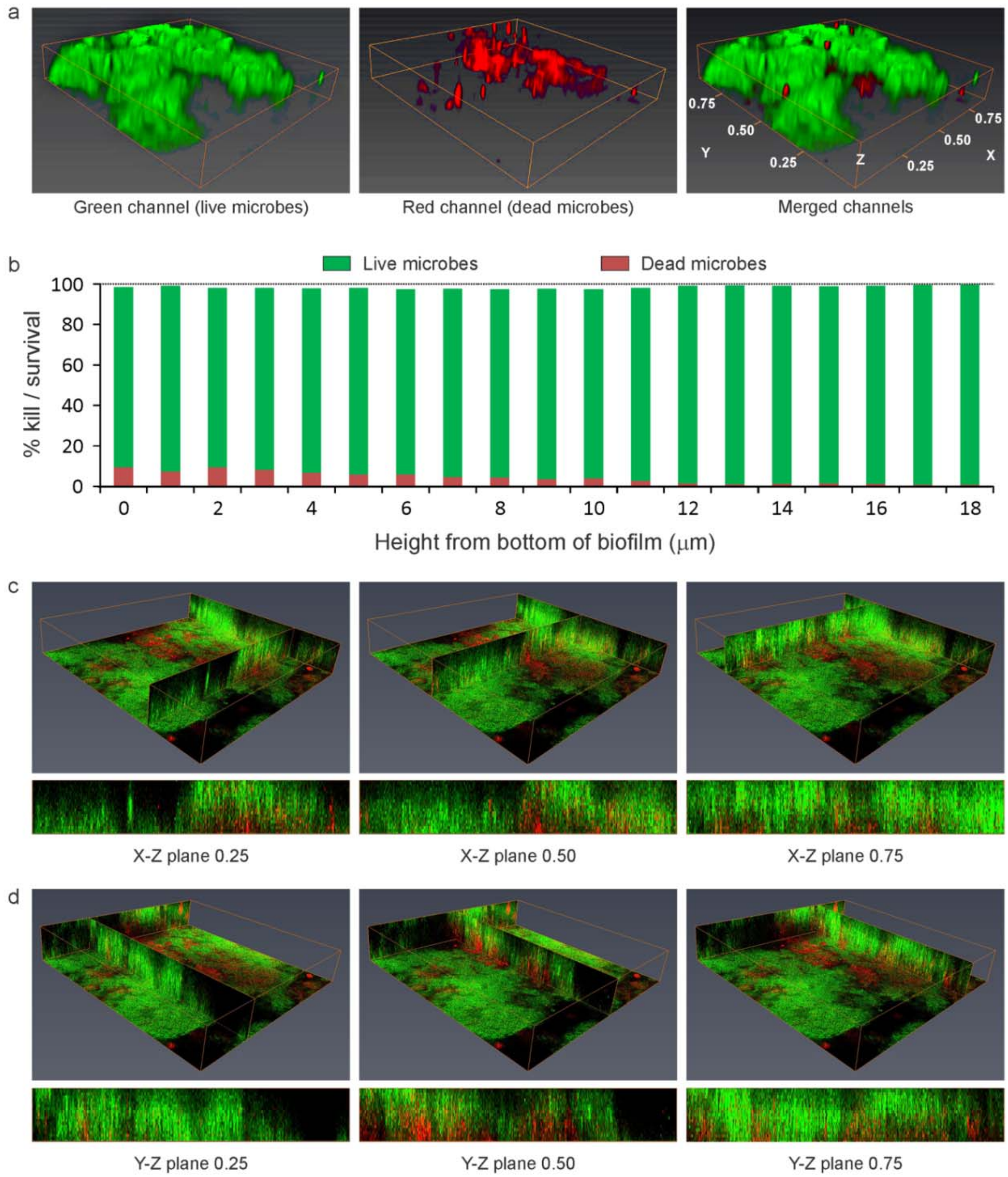

The corresponding BacLight-stained 48-hour biofilm grown on the surface of QAMS-free acrylic resin (control disk) of the subject presented in Figure 5 (Subject-25). **a.** 3-D projection for the green, red and merged channels of the biofilm (Z-axis scaled-in to the same extent as Fig.3). Green channel: live microorganisms; red channel: dead microorganisms. **b.** Live/dead microorganism distribution from the bottom to the top of the biofilm, indicating the percentage of kill in different layers of the biofilms. **c.** Perspectives of the biofilm viewed from the X-Z plane at 0.25, 0.50 and 0.75 width of the Y-axis. **d.** Perspectives of the biofilm viewed from the Y-Z plane at 0.25, 0.50 and 0.75 width of the X-axis.
